# Extracts from Observations on Exhaustion from the Effects of Heat

**Published:** 1854-09

**Authors:** H. S. Swift

**Affiliations:** Resident Physician of the New York Hospital


### RECORD OF MEDICAL SCIENCE.

*Extracts from Observations on Exhaustion from the Effects of Heat.* (*Coup de soliel.*) By H. S. Swift, M.D., Resident Physician of the New York Hospital.—The premonitory symptoms are usually slight, and of short duration. A laborer may, perhaps, have been employed until a late hour the previous night, and the next morning complains of a slight headache and a general feeling of languor. He takes his breakfast with less relish than usual, but resumes his ordinary duties. But, in the great majority of cases, even these slight symptoms are wanting. They are suddenly seized, while in the performance of their labors, with pain in the head, and a sense of fullness and oppression in the epigastrium, occasionally nausea and vomiting, general feeling of weakness, especially of the lower extremities, vertigo, dimness of vision, and insensibility. Surrounding objects appear of uniform color. In a great majority of cases, this was, so far as could be ascertained, blue or purple. In one instance every thing appeared red; in another green; and in another white. One stated that objects retained their natural color, but expressed them as being very beautiful, while to another everything appeared greatly magnified.

This may be regarded as the first stage of the disease. It is usually of short duration. In the milder forms of the disease the stupor is only momentary. The patient is at first, perhaps, aroused with difficulty, but he gradually regains his consciousness. If, however, the attack is severe, the patient shortly passes into a state of coma. The skin is hot and pungent to the touch, and by actual experiment, according to the observations of Dr. Dowler, the temperature is elevated to 112° Fahr. The pupils are dilated and insensible to light; the breathing hurried and labored; the pulse is sometimes slow and full—sometimes frequent and feeble, though the action of the heart may continue inordinately strong up to the last moment of life.

In the third stage, the symptoms are those of collapse. The pulse becomes more frequent and feeble; the respiration, which at first was hurried and labored, now becomes stertorous, and accompanied with sighing and moaning; the skin cool, or the surface of the body may retain its natural temperature, though the head may be hot; the sphincters become relaxed; extremities cold; the countenance swollen and livid; the pupils may be dilated, but are often firmly contracted; tracheal râles appear; either the patient is quiet, as if completely paralyzed, or else convulsions, often violent in character, supervene, and he dies suddenly, or he may remain in this condition for several hours.

The total number of cases admitted to this Hospital since 1845, is 150, of which 78 died. The mortality of the cases admitted in 1853 is 33 in 67.

The mortality of hospital practice must be greater than that in private, as very many were admitted in a moribund condition, and died before any treatment could be adopted, while others were rendered hopeless by being brought a long distance several hours after the attack.

The prognosis will depend on the stage of the disease. In the first stage, the prognosis is usually favorable; much, however, will depend upon the treatment adopted. The symptoms indicating collapse are always unfavorable. In 33 fatal cases the pupils were contracted in 20, moderately dilated in 7, and markedly so in 6; while, in the successful ones, the pupils were dilated in 19, and nearly natural in 15. No case recovered in which the pupils were contracted. Mere stertorous breathing is not necessarily fatal; but after the respiration becomes *sighing* and *moaning*, the prognosis is very unfavorable; only two patients recovered after this character of the breathing was present.

To these two symptoms—the condition of the pupil and the character of the respiration—I attach much value; and if other observations shall confirm this, they will furnish the most reliable basis for prognosis.

The respiration was sighing or moaning in 31 of the 33 fatal cases; convulsions were noticed in 24. This is a grave symptom, but 6 recovered after they were present. The pulse alone is no safe criterion of the actual condition of the patient, for it may continue of fair strength throughout the whole course of the disease, with no perceptible alteration either in force or frequency, though the patient may be under the free use of stimulants. This will frequently surprise those who are unaccustomed to observe it.

The pathology of this disease it too obscure and uncertain, and observation too limited, to arrive at any satisfactory conclusions in regard to the *treatment.* It is at best empirical. We regard the disease as one of debility, and we partialy treat it as such.

The great practical point to be regarded in the treatment is, that this affection is entirely distinct from *coup de soleil*, as generally understood by the term. It is a disease of "debility," and not one of "repletion." Depletion is generally contra-indicated, and stimulants are usually required.

The plan of treatment usually adopted is to place the patient in a hot bath, rendered stimulating perhaps by mustard or capsicum—or counter-irritation to the whole body by means of mustard; a stimulating enema of tr. aloes c., or, what is preferable, spts. terebinth; ice to the head *when the temperature is elevated*; brandy and tr. capsici, or even carb. ammonia if required.

The indiscriminate use of *cold effusions* is productive of harm. Injurious and often fatal effects result from them. It is a popular and erroneous idea that a patient, as soon as he is attacked, should be completely deluged with cold water. To employ it in every case would be as absurd as in cases of collapse from any cause.

Another important consideration in the treatment of the earlier stages is *rest.* In crowded cities, to which this disease is mostly confined, this caution is too much disregarded. As soon as a patient is attacked, he should be placed in a horizontal position, in as cool a place as possible, and perfect rest required. Nothing can be more serious for a patient in this condition, than to be carried, as is too often the case, upon an ordinary cart for a long distance, or allowed to remain exposed to the influence of the sun.—*From the New York Journal of Medicine.*

